# Characterization of Thermo-Mechanical and Chemical Properties of Polypropylene/Hemp Fiber Biocomposites: Impact of Maleic Anhydride Compatibilizer and Fiber Content

**DOI:** 10.3390/polym15153271

**Published:** 2023-08-01

**Authors:** Pedro Henrique Poubel Mendonça da Silveira, Mônica Cristina Celestino dos Santos, Yago Soares Chaves, Matheus Pereira Ribeiro, Belayne Zanini Marchi, Sergio Neves Monteiro, Alaelson Vieira Gomes, Neyda de La Caridad Om Tapanes, Patricia Soares da Costa Pereira, Daniele Cruz Bastos

**Affiliations:** 1 Department of Materials Science, Military Institute of Engineering-IME, Praça General Tibúrcio, 80, Urca, Rio de Janeiro 22290-270, Brazil; yago_soares2@ime.eb.br (Y.S.C.); mpmatheusr@gmail.com (M.P.R.); belayne@ime.eb.br (B.Z.M.); snevesmonteiro@gmail.com (S.N.M.); alaelson@ime.eb.br (A.V.G.); 2 Department of Materials, Rio de Janeiro State University, West Zone Campus —UERJ-ZO, Avenida, Manuel Caldeira de Alvarenga, 1203—Campo Grande, Rio de Janeiro 23070-200, Brazil; monica.santos@uerj.br (M.C.C.d.S.); neyda.tapanes@uerj.br (N.d.L.C.O.T.); patricia.soares.pereira@uerj.br (P.S.d.C.P.); daniele.bastos@uerj.br (D.C.B.)

**Keywords:** hemp, *Cannabis sativa*, polypropylene, Izod impact, FTIR, XRD

## Abstract

This article presents a comprehensive study on the physical, mechanical, thermal, and chemical properties of polypropylene (PP) composites reinforced with hemp fibers (HF) and compatibilized with maleic anhydride (MAPP). The composites were processed using a twin-screw extruder, followed by hot compression at 190 °C. Subsequently, the composites were analyzed using Izod impact and Shore D hardness tests to evaluate their mechanical properties. Thermal properties were investigated through differential scanning calorimetry (DSC) and thermogravimetric analysis (TGA), while X-ray diffraction (XRD) and Fourier-transform infrared spectroscopy (FTIR) were employed to study their chemical properties. Additionally, a statistical analysis was conducted to compare the average results of the impact and hardness tests. XRD analysis revealed that the addition of HF and MAPP led to the disappearance of peaks corresponding to the beta phase in pure PP. Hemp fibers exhibited an impressive crystallinity of 82.10%, surpassing other natural fibers, and had a significant molecular orientation angle (MFA) of 6.06°, making them highly desirable for engineering applications. The crystallite size was observed to be relatively large, at 32.49 nm. FTIR analysis demonstrated strong interactions between the fiber, compatibilizing agent, and polymer matrix. TGA tests showed that the addition of 5 and 10 wt.% MAPP resulted in complete degradation of the composites, similar to pure PP. DSC analyses indicated a reduction in crystallinity (X_c_) due to the incorporation of HF and MAPP. Shore D hardness tests revealed an increase in hardness with the addition of 5 wt.% MAPP, while a steep decline in this property was observed with 10 wt.% MAPP. In terms of impact resistance, fractions of 3 and 5 wt.% MAPP in the composites exhibited improved performance compared to the pure polymer. Analysis of variance (ANOVA) was employed to ensure the statistical reliability of the mechanical test results. This comprehensive study sheds light on the diverse properties of PP composites reinforced with hemp fibers and compatibilized with MAPP, emphasizing their potential as sustainable materials for engineering applications. The results contribute to the understanding of the structural and functional aspects of these composites, guiding future research and developments in the field.

## 1. Introduction

Global awareness of environmental and sustainability issues is increasing, striving to adapt to new concepts such as green chemistry and the green industrial revolution. These concepts have driven a series of research in the field of biocomposites [1]. Unlike composites with synthetic reinforcements, biocomposites can be disposed of and converted into ecological compost at the end of their useful life. These green materials have received considerable attention due to their enhanced properties, which give them great potential for use in various applications, including sports equipment and materials, automotive industry, construction, and even military production [2,3,4,5,6,7].

Regarding the development of biocomposites, the use of natural fibers has received increasing attention in recent years owing to their easy availability, low density, and high mechanical properties. These characteristics make natural fibers an attractive option for the manufacture of biocomposites, aligning with the search for sustainable and environmentally friendly solutions [8]. The incorporation of natural fibers in biocomposites has demonstrated significant benefits, providing improved properties and reducing environmental impact when compared to conventional composites. This growing trend in the use of natural fibers is in line with the global push towards a more sustainable economy and the industrial adoption of green materials [9,10,11,12,13,14,15].

However, despite the numerous advantages associated with the use of natural fibers, a significant limitation lies in their hydrophilic nature due to the presence of strongly polarized hydroxyl groups in their lignocellulosic structure. This characteristic renders vegetable fibers incompatible with hydrophobic polymeric matrices, resulting in weak interfacial adhesion between the fiber and the matrix [16,17]. Interfacial adhesion plays a critical role in determining the final mechanical properties of composite materials. Therefore, it is crucial to investigate various surface treatment methods that can improve the interfacial adhesion between natural fibers and polymeric matrices [18,19,20,21,22,23,24].

To solve the interfacial adhesion problem in composites, it is common to perform treatments on natural fibers in order to modify their surfaces and improve compatibility with the polymer matrix. Among the various methods used, compatibilization stands out, with maleic anhydride compatibilization being widely employed in this context [25,26]. Maleic anhydride acts as a coupling agent, reacting with the hydroxyl groups present in natural fibers and forming chemical bonds with the polymer of the matrix [27]. This chemical reaction promotes greater compatibility between the polarized surfaces of the fibers and the hydrophobic polymeric matrices, resulting in a more efficient interface [28]. Additionally, the presence of functionalized maleic anhydride in the natural fibers facilitates fiber dispersion in the matrix, which contributes to an improvement in the mechanical and physical properties of composites [29,30,31]. Thus, maleic anhydride compatibilization proves to be an effective strategy for optimizing the performance of natural fiber composites in various industrial applications.

Among the various options of natural fibers available worldwide, one of the most well-known is hemp fiber. Hemp originates from the *Cannabis sativa* L. plant, which is globally recognized and stigmatized due to one of its byproducts, marijuana. The *Cannabis sativa* L. plant, cultivated for fiber, seed, and oil extraction, is known as industrial hemp [32]. This plant species is an annual one, growing upright to a height ranging from 1 to 6 m and is predominantly dioecious [33,34].

**Figure 1:**
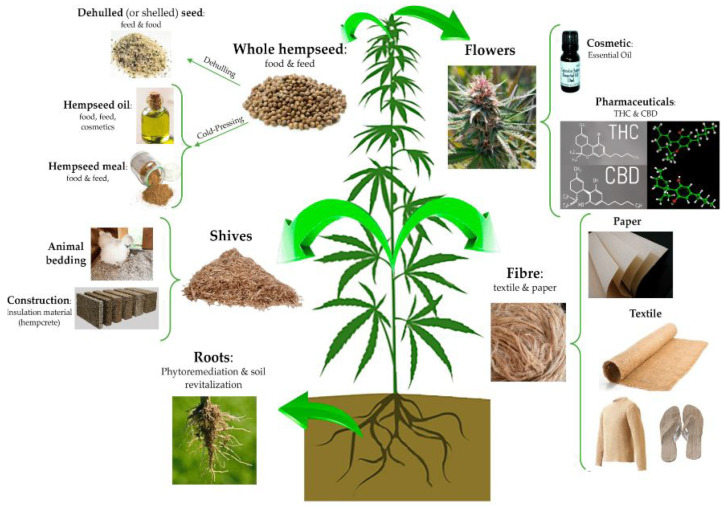
The versatile applications of the hemp plant; each part serves a specific purpose in various industries. Reprinted with permission from [47]. Copyright 2020, MDPI AG.

As shown in Figure 1, *Cannabis sativa* L. presents a series of by-products that can be used for various applications; however, one of its oldest and most utilized by-products is hemp fiber. Hemp fibers are harvested directly from the stem of *Cannabis sativa* L. plant. Their production involves mechanical separation of the fibers from the core of the bark, using a decorticator, through either retting or a combination of both methods. After separation, the dried and baled hemp fibers can undergo an additional process of mechanical separation, similar to that of cotton, which crushes them into shorter lengths and turns them into yarn. Hemp fibers obtained as a by-product of *Cannabis sativa* L. have various industrial applications [35,36,37]. Recognized for their mechanical strength, durability, and length, hemp fibers have been used for millennia in the production of ropes, paper, and textiles [38,39]. The growing global demand for natural products and sustainable and environmentally friendly systems has driven the market share of textiles, fabrics, and clothing made from hemp fiber [40]. Furthermore, hemp fiber is used in the production of biodegradable coverings, horticultural planting materials, pressed fiber products, paper and pulp, construction materials, insulation, bark-based animal bedding, bio-plastic composites, and compressed cellulose plastics [41,42,43,44,45,46].

The development of biocomposites using hemp as a reinforcing agent has attracted interest in various research studies worldwide. Similar to other natural fibers, hemp faces challenges related to low interfacial adhesion in thermoplastic matrix composites. Therefore, compatibilizers have been employed to enhance the adhesion between the matrix and reinforcement phases. Park et al. [24] conducted a study on the interfacial adhesion of hemp and jute fibers in polypropylene/maleic anhydride matrix composites (PP-MAPP), using an alkaline treatment on the fibers. The authors investigated the properties of acoustic emission and contact angle through statistical analysis. The alkaline treatment, combined with the compatibilizer used in the matrix, proved to be effective, resulting in a larger surface area and improved interfacial adhesion. This was confirmed by higher values in the contact angles due to the addition of MAPP in the composite. Sullins et al. [48] investigated the mechanical properties of hemp-fiber-reinforced polypropylene matrix composites, varying the reinforcement content between 15 and 30 wt.%, and the levels of alkaline treatment using NaOH and MAPP. Based on the results obtained, the authors observed that, in general, all the composites showed improvement in mechanical properties when treated with alkaline or MAPP. However, the composite with 5 wt.% MAPP content exhibited the best mechanical properties. Merotte et al. [49] conducted a comparative study on the mechanical properties of PP/flax and PP/hemp composites using MAPP as a grafting agent. The composites were manufactured through the hot compression method, using fabrics made from natural fibers. The results obtained revealed that, although the interfacial adhesion of hemp was higher than that of flax, the tensile strength properties were significantly lower, even after compatibilization. Paitanescu et al. [50] developed composites for application in automotive parts. They manufactured composites using a matrix made from a copolymer of polypropylene with poly[styrene-b-(ethylene-co-butylene)-b-styrene] (SEBS) and poly(styrene-b-butadiene-b-styrene) (SBS), reinforced with hemp fibers and functionalized with maleated polypropylene (MAPP). The physical, mechanical, and microstructural properties of these composites were evaluated to obtain a material with the best possible configuration for automotive component manufacturing. The characterization results indicated that the strength of the copolymers/composites with MAPP increased considerably compared to the groups without the presence of the compatibilizer. However, during the impact test, the functionalized groups showed significantly lower strength than the groups without MAPP.

With the aim of contributing to the advancement of research regarding the application of hemp fibers, this article investigates the thermal, mechanical, and chemical characterization of polypropylene matrix composites reinforced with hemp fibers and functionalized with maleic anhydride. The composites are processed using the extrusion and hot compression method, and their thermogravimetric characteristics are analyzed using TGA and DSC techniques. The interaction between the components of the composites is analyzed via FTIR. Furthermore, the formation of phases in the composites, as well as the size of crystallites and interplanar distance are analyzed through XRD. The XRD analysis also provided, for the first time, information on the microfibrillar angle of the hemp fiber, as well as the size of crystallites and crystallinity index, thus enabling evaluation and comparison of the fiber characteristics with the literature. The morphology of the composites was analyzed by SEM, and the mechanical properties of the composites were evaluated through Shore D hardness and Izod impact testing, with the results validated through analysis of variance (ANOVA).

## 2. Materials and Methods

### 2.1. Raw Materials

The compatibilizing agent used in this study was polypropylene grafted with maleic anhydride (MAPP) obtained in pellet form (Polybond^©^ 3200, Crompton Corporation, Middlebury, CT, USA). The MAPP has a melt flow index of 115 g/10 min at 190 °C/2.16 kg, a density of 0.91 g·cm^−3^, and a melting point of 160–170 °C. The virgin polypropylene (PP) used in this work was purchased from Braskem S/A (Polypropylene H 301). The PP has a molecular weight of 470,000 g/mol, a density of 0.905 g·cm^−3^, and a melt flow index of 10 g/10 min at 230 °C/2.16 kg. The hemp fibers were obtained in the form of fabric from the manufacturer Kayaan (Curitiba, Paraná, Brazil). Before processing the composites, the fibers were disentangled from the fabric, crushed in a knife mill, and sieved to achieve a particle size smaller than 20 mesh. Subsequently, the fibers were dried in an oven at a temperature of 70 °C for 2 h to remove moisture.

### 2.2. Composite Processing

**Figure 2:**
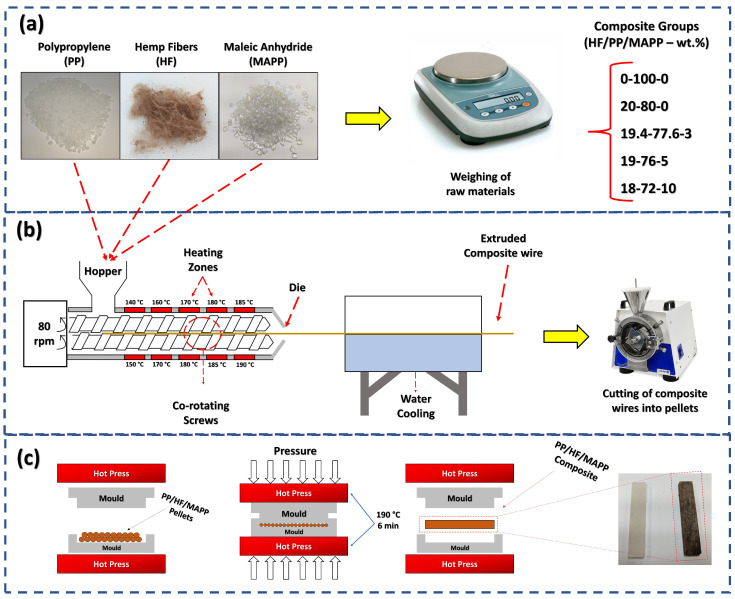
Schematic view presenting the production of HF-PP-MAPP composites: (**a**) Weighing of the starting materials; (**b**) Extrusion of the starting materials to form composite pellets; (**c**) Hot compression followed by cold compression to prepare the test specimens.

The processing steps of the composites are illustrated in Figure 2. In the first step (Figure 2a), the fibers, polymer, and compatibilizer were weighed using an analytical balance, forming 5 different groups. The first group consisted only of pure polymer, the second group contained 20 wt.% hemp fibers, and the remaining 3 groups contained varying content of hemp fibers and MAPP. After weighing, the starting materials were placed into the extruder, starting from the hopper depicted in Figure 2b. The composites were processed using a twin-screw extruder (TeckTril, DCT model) with 10 temperature zones ranging from 140 to 190 °C, rotating at 80 rpm from the feed to the die. After homogenization and heating inside the extruder, the composites emerged in the form of filaments, which were cooled in a water tank at room temperature and then cut into pellets using a knife mill. Such a procedure for producing thermoplastic composites using natural fibers is reported in the literature, where polypropylene is processed with various fibers, such as hemp [51], kenaf [52], jute and short pine needles [53], and catole coconut [54].

The test specimens for hardness and impact tests were produced using the hot compression method (Figure 2c), using equipment from the manufacturer Marconi. The pelletized composites were compressed at 190 °C for 360 s under a pressure of 6 tons. Subsequently, the mold with the samples was removed and compressed in a press at room temperature for 180 s. The described processing method was chosen for its efficiency in creating a homogeneous and well-dispersed composite. The resulting composites were characterized for their mechanical and physical properties.

Table 1 presents the compositions of the composites used in this study, along with their respective nomenclatures.

### 2.3. Characterization

#### 2.3.1. X-ray Diffraction (XRD)

To perform the XRD analysis, the fibers were cut 60 mm wide and mounted in parallel on a monocrystalline silicon substrate. The analysis was performed using the Xpert Pro MRD System equipment from PANalytics with Cobalt Kα radiation (1.789 A), at a scan speed of 4°/min and a power of 40 mA × 40 kV and scanning from 5° to 40°. With XRD analysis, we can obtain the diffraction profile of the hemp fiber in natura and thus determine parameters such as the crystallinity index (CI) and microfibril angle (MFA).

The method proposed by Segal et al. [55] was used to calculate the crystallinity index (I_c_). The value is found from the relationship that uses the intensity of the peak (002) considered as the crystalline peak and the intensity of the amorphous part (101) according to Equation (1).

(1) $$CI=\frac{I_{002}\;-\;I_{101}}{I_{002}}\;\cdot \;100\%$$

The determination of the microfibril angle (MFA) is performed through derivatives of the Gaussian curve corresponding to the peak of the (002) plane. The Origin Pro software was used to obtain the Gaussian curve, following several steps to determine the T value. To do so, it is necessary to remove the baseline from the diffractogram, allowing for the determination of the Gaussian curve for the 002 plane [56,57,58]. The T value represents the angle between the line from the center of the Gaussian peak and the intersection point between the first and second derivative. With the obtained T value, Equation (2) is used to determine the MFA.

(2) $$MFA=-12.198T^{3}+113.67T^{2}-348.4T+358.09$$

To determine the crystallite size (CS), the Scherrer equation was used, given by Equation (3), which relates the size of submicrometer crystallites in a solid to the broadening of a peak within a diffractogram [59]. The calculation involves using the maximum full width at half maximum (FWHM) of the most intense peak in the diffractogram, in the case of natural fibers, the peak corresponding to the (002) plane [60,61,62].

(3) $$CS=\frac{K\cdot \lambda }{\beta \cdot cos\theta }$$

where CS represents the crystallite size, λ represents the wavelength of the Co-Kα radiation (1.789 Å), β represents FWHM fraction angles, K is the correction factor of 0.89, and θ represents the diffraction angle of the highest peak of the hemp samples.

The average interplanar distance of the composites was calculated using Equation (4), which takes into account the Miller indices obtained from the diffractograms of both PP and HF-PP-MAPP composites.

(4) $$d_{\mathrm{hkl}}=\frac{\lambda }{2\cdot sen\theta }$$

where *d*_hkl_ is the interplanar distance (nm), λ is the wavelength of CoKα (1.789 Å), and θ is the peak angle in the diffractogram divided by 2.

#### 2.3.2. Fourier Transform Infrared Spectroscopy (FTIR)

Fourier-transform infrared spectroscopy (FTIR) analysis was acquired using a Nicolet 6700 FTIR spectrometer (Thermo Fisher Scientific, Pinheiros, São Paulo, Brazil). The samples were mounted on an attenuated total reflectance (ATR) accessory equipped with ZnSe crystal prior to scanning. The spectra were obtained with an accumulation of 128 scans.

#### 2.3.3. Thermogravimetry/Derivative Thermogravimetry (TG/DTG)

The thermal stability of the extruded samples was evaluated by thermogravimetry (TG/DTG) analysis. We used a PerkinElmer STA 6000 simultaneous thermal analyzer with aluminium oxide crucible, temperature ramp from 30 to 500 °C, heating rate of 10 °C/min and N_2_ atmosphere.

#### 2.3.4. Differential Scanning Calorimetry (DSC)

DSC analysis of the extruded samples was also performed using a PerkinElmer STA 6000 simultaneous thermal analyzer with alumina pan. The samples were analyzed under N_2_, according to the following cycles: first cycle, heating from 30 to 200 °C, at a heating rate of 10 °C/min and maintenance at 200 °C for 2 min; second cycle, cooling to 30 °C at cooling rate of 10 °C/min; third cycle, same temperature range and heating rate of the first cycle (except for the 2 min isothermal); and fourth cycle, the same temperature range and cooling rate as the second cycle. We considered the data of the second heating curves.

The crystallization temperature (T_c_), the melting temperature (T_m_) and the melting enthalpy (Δ*H*^0^_m_) were obtained from the heating–cooling–heating cycle of the sample. The crystallinity, X_c_ (%), of the different composites was obtained by Equation (5).

(5) $$X_{c}=\frac{\Delta H_{m}}{\Delta H_{m}^{0}\cdot \left(1-m\right)}$$

where: Δ*H*_m_ corresponds to the theoretical melting enthalpy of the fully crystalline polypropylene, whose value according to data from the literature is 165 J/g, and w is the mass fraction of the filler and compatibilizer [63].

#### 2.3.5. Shore-D Hardness

The Shore D hardness tests were carried out according to the ASTM D2240-05 standard [64]. The measurements were performed using the Shore D Durometer (Type GS 702), which provided the Shore D hardness value of the material under investigation. To ensure the accuracy of the results, the highest and lowest values obtained from each sample were excluded, and the arithmetic mean of the remaining five determinations was calculated.

#### 2.3.6. Izod Impact Test

To evaluate the impact resistance of the processed samples, the Izod impact test was employed following the ASTM D-256 standard [65]. The test was conducted using a universal pendulum impact testing machine, with the analysis of five samples for each composition. Each sample was securely mounted in a vertical position and subjected to the impact of a pendulum with a force of 5.5 J applied at the center. This method was chosen for its widespread acceptance and reliability in assessing the impact resistance of polymeric materials.

#### 2.3.7. Statistical Analysis

The statistical analysis employed multiple linear regression to obtain mathematical models that relate the properties of the composites using polypropylene (PP), hemp fiber, and maleic-anhydride-grafted polypropylene (MAPP), aiming to optimize the mechanical performance, specifically impact strength and hardness. Analysis of variance (ANOVA) was performed to evaluate such models. The quantity of PP, HF, and MAPP in the composite was used as independent variables, also called factors. The dependent variables of the regression model used to optimize the mechanical performance of the composite were hardness (H) and Izod impact strength (I). For each of the five sample groups processed in this study, ten hardness measurements and five impact measurements were performed, totaling 50 and 25 repetitions, respectively, each one conducted randomly to reduce systematic errors. These results allowed obtaining the regression models. A second-degree polynomial model was evaluated for each response variable.

#### 2.3.8. Scanning Electron Microscopy (SEM)

Analysis of the morphology of fractured samples following an impact test was conducted using a Quanta FEG 250 microscope from Fei (Hills-Boron, CA, USA). The microscope was equipped with a secondary electron detector, operated at an accelerating voltage of 5 kV, and had a magnification range of 500×. To ensure accurate results, the fibers were coated with gold using the Leica Ace600 sputtering equipment (Wetzelar, Germany).

## 3. Results and Discussion

### 3.1. XRD Results

**Figure 3:**
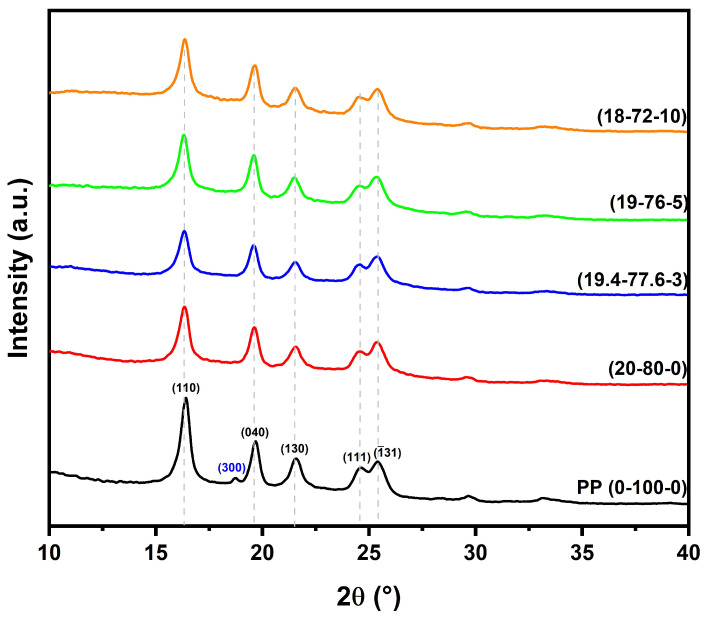
Diffractograms of HF-PP-MAPP composites.

Figure 3 shows the diffractograms of the hemp fiber and HF-PP-MAPP composites.

Figure 3 displays the diffractograms of PP and HF-PP-MAPP composites. In relation to polypropylene, five peaks were identified in the range between 15 and 30 2θ, corresponding to the α crystalline phase of PP, which has a monoclinic structure. These peaks correspond to the planes (110) at 16.42°, (040) at 19.70°, (130) at 21.60°, (111) at 24.51°, and ($\bar{1}$31) at 25.42° [66,67]. The peak corresponding to the (300) plane found at 18.72° is related to the β crystalline phase of polypropylene. The formation of the β phase occurs during the cooling of polypropylene during processing, where low cooling temperatures can induce the formation of β phase peaks [68,69].

It can be observed that the addition of hemp fibers and the compatibilizer (MAPP) resulted in the disappearance of the β phase peak, as observed in the composite diffractograms. Furthermore, the incorporation of HF and MAPP led to a reduction in the intensity of the (110), (040), and (130) peaks. The peaks corresponding to the (101) and (002) phases, related to the lignocellulosic formations of the natural fibers, did not appear in the composite diffractograms.

Table 2 presents the positions of the peaks for PP and HF-PP-MAPP composites. It can be observed that the addition of fiber and compatibilizer caused a slight shift in the peaks related to the PP phases, resulting in an increase in the average interplanar distance of the composites, as shown in Table 2, as well as an increase in the average crystallite size. PP exhibited an average crystallite size of 23.35 nm. The inclusion of reinforcement and compatibilizer resulted in a slight increase, which can be attributed to the larger crystallite size of the hemp fiber, as presented in Table 3.

The peaks related to the planes of the natural fibers were not detected in the diffractograms of HF-PP-MAPP composites due to two main factors. The first factor is related to the size of the fiber crystallites, which are larger than those of PP. This difference leads to a reduction in the intensity of the polymer peaks, as observed in Figure 3, making their detection more challenging and only possible by observing the decrease in the intensity of the polymer peaks. The second factor is the orientation of the fibers [70]. As the hemp fibers are randomly dispersed in the matrix, their distribution may not be entirely homogeneous, which can hinder X-ray detection during the analysis.

**Figure 4:**
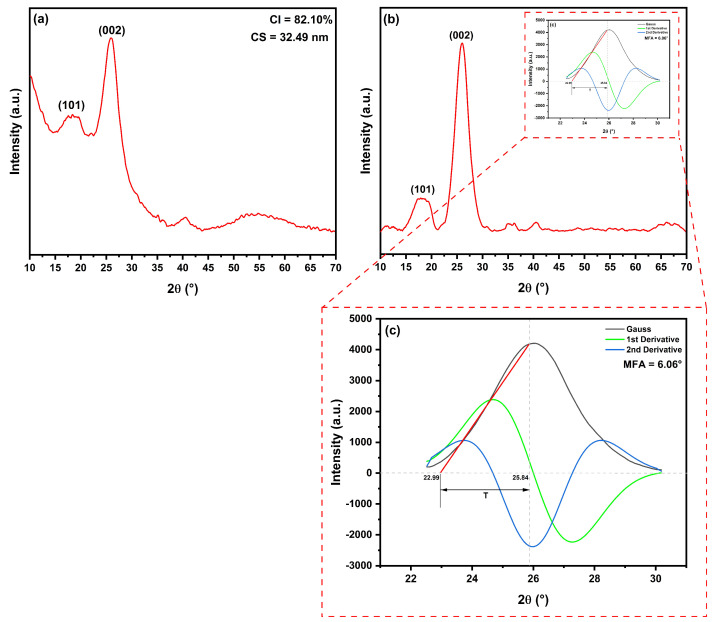
XRD results of hemp fiber: (**a**) Fiber diffractogram with peak indexing; (**b**) Fiber diffractogram after baseline correction for microfibril angle derivation; (**c**) Deconvolution result of the (002) peak and derivatives for microfibril angle determination in hemp fiber.

Figure 4 shows the diffractogram of the hemp fiber and the results of the determination of the microfibril angle in the fiber.

The diffractogram of the hemp fiber, shown in Figure 4, presented three peaks corresponding to the already known planes of natural lignocellulosic fibers: the peak of the plane (101) at 18.41° and the peak referring to the plane (002). The peak corresponding to the plane (101) occurs due to the presence of a structure composed of pectin, lignin, hemicellulose, and cellulose, while the more intense peak, relative to the plane (002), occurs in natural fibers due to the formation of α-cellulose [71,72]. The peak corresponding to the (040) plane is observed with low intensity in the diffractogram. This peak appears in the diffractogram due to the presence of cellulose in the fiber. Due to its weak intensity and the fact that this plane is not used in the calculations of crystallite size, microfibril angle, and crystallinity index, many authors choose to disregard the presence of this plane and do not index it in the diffractograms.

The crystallinity index of hemp fiber was calculated using Equation (1), and the results indicate a value of 82.10%. This high crystallinity value is attributed to a probable low content of lignin, pectin, and other lignocellulosic components. During the calculation, it is observed that the higher and broader the peak corresponding to the (101) plane, the lower the crystallinity, as it indicates a higher amount of amorphous constituents in the natural fiber. This phenomenon was observed in the work of Vijay et al. [73], resulting in a crystallinity value below 40% for *Pennisetum orientale* grass fiber. Table 3 presents a comparison of crystallinity index (CI) values, revealing that hemp fiber has significantly higher crystallinity than several other fibers, falling just slightly below carnauba fibers and Jack tree fibers, both with 86% crystallinity.

**Table 3:** Comparison of the properties Crystallinity Index (CI), Microfibril Angle (MFA), and Crystallite Size (CS) of hemp fiber obtained by XRD with other fibers reported in the literature.

Fiber	CI (%)	MFA (°)	CS (nm)
Hemp (*Cannabis sativa* L.) (PW*)	82.10	6.06	32.49
Seven-Islands-Sedge(*Cyperus malaccensis*) [58]	62.47	7.36	2.56
Carnauba (*Copernicia prunifera*) [74]	86.90	7.48	-
Ubim (*Geonoma baculífera*) [75]	63–83	7.42	-
Mendong Grass (*Fimbristylis globulosa*) [76]	58.60	22.90	14.3
Jack Tree Fiber [77]	86.00	29.00	5.19
*Heteropogon contortus* [78]	54.10	14.53	-
*Nendran Banana Peduncle* [79]	53.30	9.45–13.87	4.72
*Cereus hildmannianus* [80]	40.19	1.38	28.27
Aerial roots of banyan tree [81]	72.47	10.88	6.28
*Prosopis juliflora* bark [82]	46.00	10.64	15.00
*Sida cordifolia* stem [79]	56.92	9.50	18.00
*Thespesia populnea* [83]	48.17	13.94	3.54

Regarding microfibril angle (MFA), hemp fibers exhibited a value of 6.06°. MFA is a structural characteristic that describes the orientation of microfibrils within fibers. Essentially, MFA is the angle formed by the microfibrils in relation to the longitudinal axis of the fiber [84]. This orientation has a direct influence on the mechanical properties of natural fibers, such as strength, rigidity, and toughness. A lower microfibril angle, closer to 0°, generally results in stronger and stiffer fibers, while a higher angle, close to 90°, can increase fiber flexibility and proneness to fracture. Additionally, the variation in microfibril angle can also affect other properties of the fibers, such as moisture absorption and air permeability.

The result obtained for hemp fiber show an angle close to the axis of the plant (near 0°), as compared in Table 3, where its angle is below almost all other fibers, except for *Cereus hildmannianus* fiber, which has an MFA of 1.38°. Fibers from Brazil, such as carnauba (7.48°), ubim (7.42°), and seven-islands-sedge (7.36°), have MFA values close to hemp fiber.

The calculated crystallite size from the (002) peak was found to be 32.49 nm. This result is significantly larger than that observed in other natural fibers, as indicated in Table 3. Among all the compared fibers, only *Cereus hildmannianus* fiber, which exhibited a low MFA, showed this behavior, demonstrating a high CS value similar to that of hemp.

The parameters of CI and CS play an important role in the selection of natural fibers for specific applications due to their distinct properties. An increase in CI of natural fibers results in greater stiffness and lower flexibility [85]. CS is directly related to CI as it tends to increase as CI increases. This is because a reduction in the surface area of the crystallites corresponding to the amorphous regions of cellulose is observed [86].

### 3.2. FTIR Results

**Figure 5:**
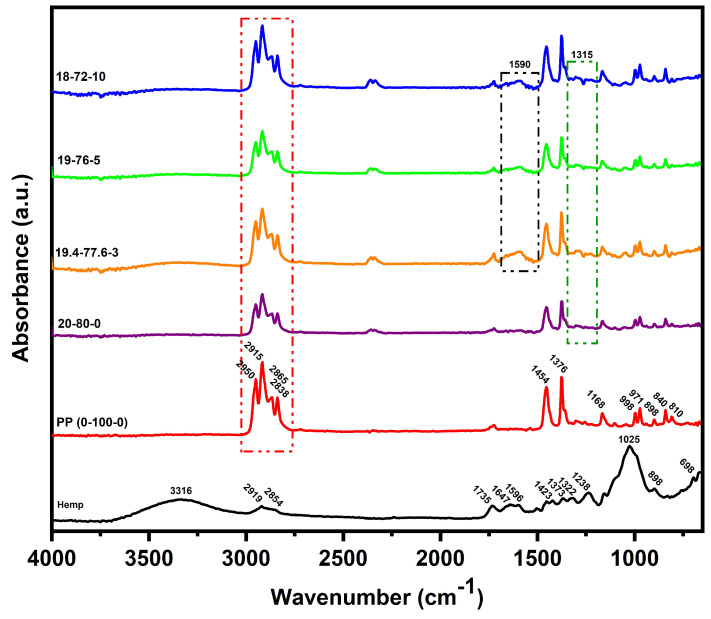
FTIR-ATR spectra of the hemp fibers and the composite samples.

The FTIR spectra of the hemp fibers and the composites are shown in Figure 5.

The FTIR in this study revealed a number of absorption peaks corresponding to the various chemical groups present in the fiber. As shown in Figure 5 and Table 4, the peaks at 2919 cm^−1^ and 2854 cm^−1^ correspond to the C-H stretching vibration of CH and CH_2_ in cellulose and hemicellulose, while the peak at 1735 cm^−1^ and 1647 cm^−1^ corresponds to the carbonyl C=O stretching vibration of ester linkage of lignin and acetyl groups of hemicellulose, and the C=C stretching of the alkene group. The peak near 1373 cm^−1^ corresponds to the CH bending of cellulose and hemicellulose [87,88,89,90].

Additionally, we found that the carbon–oxygen single bond also has an absorption in the fingerprint region, with the absorption bands at 1238 cm^−1^ and 1025 cm^−1^ attributed to the symmetrical stretching and vibration of C-O, suggesting the presence of acetyl and alkoxy groups in hemicellulose and lignin, respectively. The peak observed at 687 cm^−1^ represents C-OH out-of-plane, denoting the presence of cellulose [91,92,93,94].

The FTIR spectrum of polypropylene (0-100-0), shown in Figure 5, displays peaks at various wavenumbers indicating the presence of different functional groups. The peaks observed near 2950, 2915, 2865, and 2838 cm^−1^ correspond to the CH_2_ and CH_3_ groups. Additionally, the symmetric bending vibration of CH_3_ was observed near 1455 and 1376 cm^−1^. The stretching vibrations of CH-CH_3_ and CH-CH_3_ were observed at 1168 and 971 cm^−1^, respectively, with medium intensity peaks around 840 cm^−1^ representing C-H bonds [95,96]. In addition, the HF-PP-MAPP composites contained intense vibrations around 1320 cm^−1^, attributed to C-H stretching, and at 1590 cm^−1^ confirming the presence of COO- and assigned to stretching of the carboxyl group present in maleic anhydride [95,96]. The spectra of all the composites showed bands corresponding to PP, MAPP, and hemp fibers, confirming the physical interaction between the elements of the composites.

These results provide important insights into the chemical composition of the hemp fibers, which can be useful in optimizing the properties and processing of hemp-based composites. The presence of various functional groups in the hemp fibers can potentially influence the interfacial interaction between the fibers and the polymer matrix in the composite, and may play a role in determining the mechanical and physical properties of the final product.

### 3.3. Thermal Analysis

**Figure 6:**
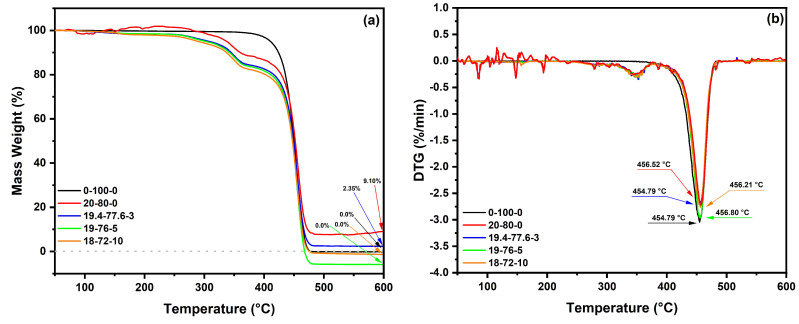
Comparative graphs of thermogravimetric analysis of HF-PP-MAPP composites: (**a**) TG curves; (**b**) DTG curves.

The thermal decomposition of PP and HF-PP-MAPP composites was investigated using thermogravimetry (TG). Figure 6a illustrates the weight loss versus temperature curves, while Figure 6b displays the corresponding first-derivative curves.

Moreover, Table 5 presents important thermal parameters including the degradation onset temperature (T_onset_), the maximum degradation temperature (T_max_), the temperature of degradation endset (T_endset_) and the degradation percentages at different stages of degradation during TG/DTG analysis.

It is possible to observe, in the TG curves of Figure 6a, that the thermal degradation of the composites occurred in two distinct stages. The first stage, which started at room temperature and was completed at 200 °C, is associated with moisture loss. In the case of pure polypropylene, there was a mass loss of only 0.27%. However, after adding 20 wt.% hemp fibers (20-80-0), no degradation was observed up to 200 °C, as illustrated in Figure 6a and Table 5. On the other hand, the addition of a compatibilizer resulted in an increase in degradation during the first stage. In the composites (19.4-77.6-3), (19-76-5), and (18-72-10), degradation values of 1.51, 1.49 and 2.03% were observed, respectively.

The second stage of degradation begins when the test reaches a temperature of 200 °C. In this stage, there is a sharp increase in the degradation curve, and the actual onset of degradation (T_onset_) occurs at 413 °C in the 0-100-0 sample. The addition of HF and MAPP resulted in a delayed onset of T_onset_, with temperatures for the composite without MAPP (20-80-0) and the composites with MAPP (77.6-16.4-3, 19-76-5, and 18-72-10) ranging between 423 and 425 °C. The peak degradation temperature (T_max_) was recorded in Figure 6b, showing that the addition of the fiber without a compatibilizer and with 5 and 10% wt.% MAPP caused a slight delay in T_max_, while the composite with 3 wt.% MAPP showed no temperature difference at this point. The temperature representing the end of the second stage of degradation (T_endset_) showed minimal changes. The temperature range varied between 472 and 474 °C. At this point in the test, the 19-76-5 sample already exhibited 100% degradation. At the end of the test at 600 °C, both pure polypropylene and the 18-72-10 composite reached maximum degradation, while the composites 20-80-0 and 19.4-77.6-3 had residue contents of 9.10 and 2.35%, respectively.

**Figure 7:**
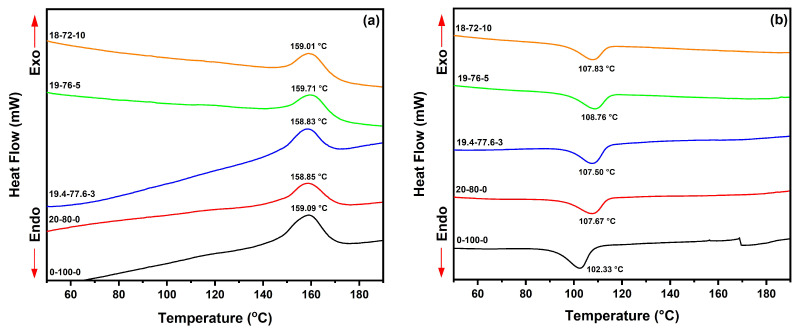
DSC thermograms of HF-PP-MAPP composites: (**a**) Second heating cycle; (**b**) First cooling cycle.

Figure 7a,b show the main thermal transitions of PP and HF/PP/MAPP composites recorded by DSC analysis.

Figure 7a displays the thermograms of the composites during the second heating cycle, while Figure 7b shows the curves during the first cooling cycle. From the thermograms, it was possible to determine the crystallization temperature (T_c_) during the first cooling cycle. Sample 0-100-0 recorded a T_c_ of 102 °C during cooling. The addition of hemp fiber and compatibilizer resulted in an increase in T_c_, between 107 to 108 °C.

The T_c_ of the composites obtained in this study was lower compared to other research findings presented in the literature. Burgada et al. [63] achieved T_c_ values approximately 10 °C higher, ranging from 115 to 118 °C, when using recycled polypropylene (rPP) matrix composites reinforced with hemp and MAPP. Stelea et al. [97] processed PP composites reinforced with different fractions of hemp and obtained a T_c_ temperature in the range of 115 °C during cooling cycle.

The use of HF combined with MAPP compatibilization did not result in significant changes in the melting temperature (T_m_) of HF-PP-MAPP composites. The thermal transition results of the composites are presented in Table 6. The 0-100-0 sample exhibited a T_m_ of 159 °C, while the 20-80-0 and 19.4-77.6-3 composite samples showed T_m_ with values of 158 and 159 °C, respectively. The incorporation of HF in the composites led to a decrease in the fusion enthalpy (ΔH_m_). Additionally, MAPP compatibilization resulted in a considerable reduction in ΔH_m_, consequently decreasing the crystallinity values (X_c_) of the composites. PP naturally exhibited the highest crystallinity value, with 42.89% crystalline fraction. These results are consistent with the study by Tanjung et al. [98], who reported slightly lower values (38%) for pure polypropylene and a reduction in X_c_ following the addition of chitosan and alkali treatment. On the other hand, Salazar-Cruz et al. [99] observed a slight increase in crystallinity of the composites with the addition of pistachio shell particles, resulting in an increase in X_c_ from 37% for pure polypropylene to 41% in the composite with 2 phr of fibers.

The addition of fibers reduced the crystallinity of the composites, with the crystallinity of the 20-80-0 composite being X_c_ = 39.89%, while the 19.4-77.6-3, 19-76-5, and 18-72-10 composites exhibited 34.97, 38.76 and 23.87%, respectively.

### 3.4. Mechanical Properties: Hardness and Impact Resistance

In Table 7, a summary of the Shore D hardness and impact resistance results of the composites is provided.

Based on the hardness results presented in Table 7, it is evident that incorporating 5 wt.% compatibilizer led to a 5.89% increase in this property compared to virgin PP. This can be attributed to improved uniformity in the distribution of components in the matrix brought about by MAPP [95,96,100].

Interestingly, the composite containing 10 wt.% MAPP exhibited the lowest hardness among all the groups, measuring at 58.4 Shore D. This can be explained by the possibility that an increase in the coupling agent up to 10 wt.% may have caused saturation of MAPP in the composite.

Furthermore, the formulations 20-80-0 and 19.4-77.6-3 showed comparable hardness values (62.7 and 61.2 Shore D, respectively), which were closer to the values observed for the PP matrix [96]. The standard deviation also helped mitigate any discrepancies in the results.

The impact strength results demonstrate that the impact resistance of the 0-100-0 sample in this study, measured at 24.32 ± 5.61 KJ/m^2^, as consistent with the literature findings [15,21,96]. However, the impact strength decreased as the amount of hemp fiber added to the formulations increased compared to pure PP. This reduction in impact strength could be attributed to the hydrophilic nature of natural fibers, which negatively impacted the adhesion between the matrix and the reinforcement phase of the composites. Consequently, SEM analysis revealed filler debonding, filler fracture, undesirable agglomeration, and filler pull-out.

Among the various composites, 20-80-0 and 19.4-77.6-3 exhibited slightly higher impact resistance when a 3 wt.% compatibilizer was used, most likely due to the equilibrium between chain scission and self-reinforcement mechanisms attributed to the presence of MAPP in the formulations [95,96,100]. However, when 10 wt.% coupling agent was added, the reduction in impact resistance was more pronounced than with the addition of 3 or 5 wt.% MAPP, which was consistent with the SEM images and hardness results.

### 3.5. Statistical Analysis

**Figure 8:**
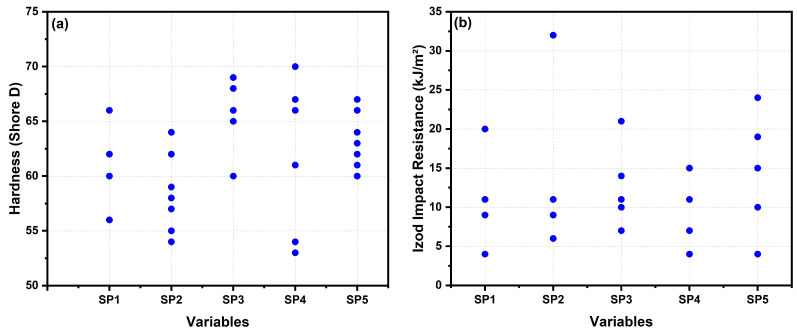
Values of response variables for (**a**) hardness and; (**b**) Izod impact resistance of the specimens (SP1-SP5) according to the experiment matrix.

The hardness (H) and Izod impact strength (I) responses are depicted in Figure 8. ANOVA results, presented in Table 8 and Table 9, were employed to identify the factors and their interactions that significantly influenced the mechanical performance of the composite. This study considered second-degree interactions such as PP-HF, PP-MAPP, and HF-MAPP.

Table 8 and Table 9 present the *p*-values for the factors (PP, HF, MAPP) and their interactions, which determine their influence on the response variables (H and I). A confidence level of 95% was considered. If the *p*-value of a factor or interaction is equal to or lower than the significance level (5%), it indicates a significant correlation between the response variables and the factor. Conversely, *p*-values higher than 0.05 indicate the absence of correlation.

The results presented in Table 8 highlight the statistical significance of the PP factor and the PP-HF and PP-MAPP interactions concerning the response variable H, confirming that the PP content has a significant influence on composite hardness. However, the regression results indicate that the impact resistance is unaffected by the presence of HF. The mean values of Shore D hardness show low variation, along with a low standard deviation, making them statistically similar.

The regression model for the response variable H has an adjusted R-Sq (adj) value of 92.96%, indicating a high level of predictability. This value exceeds the 90% threshold, demonstrating that the model performs well in predicting the hardness of the composite. On the other hand, the adjusted R-Sq (adj) for the response variable I is relatively low at 68.67%, suggesting the possibility of other factors influencing the impact resistance. From this low R-Sq (adj) value, it can be inferred that the means of the PP group and the HF-PP-MAPP composites are statistically different, likely due to significant variation in impact resistance resulting from the addition of reinforcement and compatibilizer.

To ensure the validity of the regression models and verify the assumptions of ANOVA, a residual analysis was conducted. Equations (6) and (7) represent the models’ equations for the response variables H and I, respectively.

(6) H=0.628·PP+0.0057·PP·H+0.166·PP·MA±4.53

(7) I=0.1155·PP−0.2835·PP·MA±6.71

### 3.6. Microstructural Analysis

**Figure 9:**
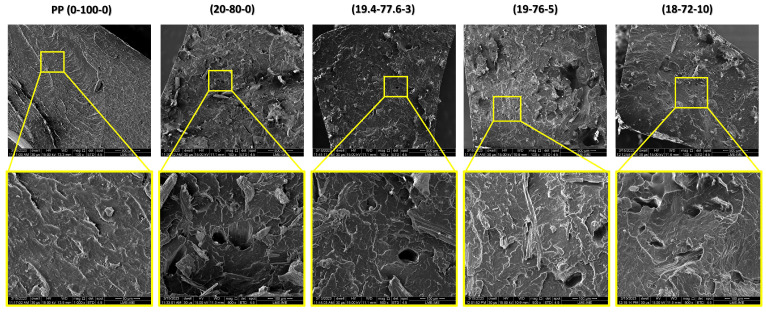
Micrographs showing fracture surfaces of the composites after Izod impact testing at magnifications of 100× and 500×.

Figure 9 shows SEM images obtained from composites fractured by the Izod impact test.

SEM micrographs were obtained to analyze the fracture surface of the composites after the Izod impact test. It was observed that the 0-100-0 sample exhibited ductile fracture marks on its surface, revealing the mechanical behavior of pure polypropylene. In contrast, the sample with the addition of 20 wt.% hemp fibers (20-80-0) showed signs of low interfacial adhesion, as evidenced in Figure 9, where fiber displacement is observed. On the other hand, the composites with 3 and 5 wt.% MAPP (19.4-77.6-3 and 19-76-5) exhibited uniformly dispersed fibers that were well incorporated into the polymer matrix with minimal surface defects.

These results demonstrate that the addition of up to 5 wt.% MAPP is effective in improving the performance of PP/hemp composites, which is supported by the Izod impact test results described in Table 5. This indicates that compatibilization in these fractions improved the mechanical performance of the fiber compared to the composite without MAPP. The strong fiber–matrix interface, especially in the presence of MAPP, is expected to enhance the mechanical, thermal, and physical properties, as reported in the literature [44,101].

Finally, the micrographs of the 18-72-10 sample showed that the addition of 10 wt.% compatibilizer is excessive, acting as a plasticizer in the polymeric matrix and reducing the mechanical performance of the composite. Both the 18-72-10 and 19-76-5 samples exhibited internal defects such as pores, which may be attributed to the accumulation of gases within the composite during hot compression processing.

## 4. Summary and Conclusions

In this study, composites were produced using a polypropylene (PP) matrix reinforced with hemp fibers (HF) and compatibilized with maleic anhydride (MAPP). The physical and mechanical properties of the composites were evaluated through Izod impact tests and Shore D hardness tests. Thermal properties were investigated using DSC and TGA, while chemical properties were analyzed through XRD and FTIR. Additionally, a statistical analysis comparing the average results of the impact and hardness tests was conducted. Based on the obtained results, it is possible to conclude that:

- XRD results indicated the presence of peaks corresponding to the beta phase in PP, which disappeared in the composites with the addition of HF and MAPP. The addition of HF and MAPP to the composites caused an increase in the average interplanar distance and crystallite size, as the hemp fiber’s crystallite size is larger than that of PP.
- HF exhibited a crystallinity index of 82.10%, significantly higher compared to other natural fibers. Moreover, the microfibril angle (MFA) of 6.06° makes the fiber an attractive material for engineering applications. The crystallite size was considered high, with a value of 32.49 nm.
- FTIR analyses revealed interactions between PP, HF, and MAPP, with noticeable peaks related to MAPP compatibilization increasing as the compatibilizer content increased.
- TGA tests demonstrated that the addition of 5 and 10 wt.% MAPP resulted in complete degradation of the composite, similar to PP. Despite the complete degradation of the composites with these MAPP concentrations, the T_onset_, T_max_, and T_endset_ temperatures showed minimal variation, indicating similar thermal behavior of the HF-PP-MAPP composites.
- DSC analyses revealed a reduction in crystallinity (X_c_) as a result of incorporating HF and MAPP. Similar to the TGA results where degradation temperatures remained mostly unchanged, DSC showed minimal variation in T_c_ temperatures, with the only significant variation observed from PP to the 20-80-0 composite. As for T_g_, no variation occurred due to the addition of HF and MAPP.
- Shore D hardness tests indicated an increase in hardness with the addition of 5 wt.% of MAPP in the composite, but a sharp decrease in this property was observed with 10 wt.%.
- Izod impact tests showed that 3 and 5 wt.% fractions of MAPP in the composites improved impact resistance compared to composites without MAPP, while the addition of 10 wt.% MAPP critically reduced the composites’ impact strength.
- Variance analysis (ANOVA) was performed to verify the statistical reliability of the mechanical test results, subsequently indicating that the hardness test results are statistically similar, while the impact test results are statistically different.

## Figures and Tables

**Table 1:** Composition of the studied composites as a function of hemp fiber, polypropylene, and maleic anhydride content.

Sample (HF-PP-MAPP)	Hemp Fiber (HF—wt.%)	Polypropylene (PP—wt.%)	Maleic Anhydride (MAPP—wt.%)
0-100-0	0	100	0
20-80-0	20	80	0
19.4-77.6-3	19.4	77.6	3
19-76-5	19	76	5
18-72-10	18	72	10

**Table 2:** Positions and intensities of peaks, Average interplanar distance, and Crystallite size of PP and HF-PP-MAPP composites.

Sample	(1 1 0)		(0 4 0)		(1 3 0)		(1 1 1)		($\bar{1}$31)		d_hkl_ (Å)	CS (nm)
	2θ (°)	I_(1 1 0)_	2θ (°)	I_(0 4 0)_	2θ (°)	I_(1 3 0)_	2θ (°)	I_(1 1 1)_	2θ (°)	I_($\bar{1}$31)_		
0-100-0	16.42	8941	19.70	5923	21.60	4705	24.51	4073	25.42	4504	4.9094 ± 0.8869	23.35 ± 5.37
20-80-0	16.38	6939	19.64	5495	21.59	4145	24.46	3780	25.36	4456	4.9496 ± 0.8916	25.35 ± 5.38
19.4-77.6-3	16.35	5916	19.60	4909	21.59	3733	24.43	3576	25.43	4139	4.9227 ± 0.8977	24.25 ± 3.68
19-76-5	16.33	7449	19.62	6030	21.51	4465	24.51	3902	25.45	4499	4.9235 ± 0.9029	24.75 ± 5.69
18-72-10	16.37	7880	19.67	6047	21.55	4489	24.47	3830	25.46	4392	4.9168 ± 0.8951	23.66 ± 4.06

**Table 4:** Funcional groups present in hemp fibers.

Wavenumber (cm^−1^)	Functional Group	Compound
2919	Stretching vibration of CH	Cellulose
2854	Stretching vibration of CH_2_	Hemicellulose
1735	C=O stretching vibration	Lignin and hemicellulose
1647	C=C stretching	Alkene group
1373	CH bending	Cellulose and hemicellulose
1238	C-O stretching vibration	Hemicellulose
1025	C-O stretching vibration	Lignin
698	C-OH out-of-plane	Cellulose

**Table 5:** Thermal parameters obtained by TGA of the HF-PP-MAPP composites.

Sample (wt.%) HF-PP-MAPP	Mass Loss			T_onset_ (°C)	T_max_ (°C)	T_endset_ (°C)
	At 200 °C (%)	End of Second Stage (%)	At 600 °C (%)			
0-100-0	0.27	98.91	100	413	455	473
20-80-0	0.00	90.75	90.90	425	456	474
19.4-77.6-3	1.51	96.01	97.65	424	455	474
19-76-5	1.49	100.00	100	423	457	474
18-72-10	2.03	99.02	100	423	456	472

**Table 6:** Thermal transitions obtained from DSC thermograms of the HF/PP/MAPP composites.

Sample (wt.%) HF-PP-MAPP	T_c_ (°C)	T_m_ (°C)	Δ_Hm_ (J/g)	X_c_ (%)
0-100-0	102	159	70.77	42.89
20-80-0	107	158	52.66	39.89
19.4-77.6-3	107	158	44.78	34.97
19-76-5	108	159	48.61	38.76
18-72-10	107	159	28.36	23.87

**Table 7:** Results of Shore D hardness and impact resistance of the studied composites.

Sample (wt.%) HF-PP-MAPP	Hardness Shore D	Impact Strength (kJ/m^2^)
0-100-0	62.80 ± 3.29	24.32 ± 5.61
20-80-0	62.70 ± 2.31	10.77 ± 0.86
19.4-77.6-3	61.20 ± 3.77	13.79 ± 3.43
19-76-5	66.50 ± 2.63	12.21 ± 2.78
18-72-10	58.40 ± 3.73	5.51 ± 1.32

**Table 8:** ANOVA of factorial design for the hardness (H).

Source of Variation	Degree of Freedom (DF)	Sum of Squares (SQ)	Mean Squares (MQ)	F	Significance of F
Regression	6	194,315	32,386	2366	1.7 × 10${}^{-52}$
Residue	46	944	20		
Total	52	195,260			
**Term**	**Coeficients**	**Standard Error**	**Stat t**	**Value-** ***p***	
PP	0.6280	0.0143	43.8293	0.0000	
HF	0.0000	0.0000	65,535	0.5	
MAPP	−10.9853	5.5895	−1.9654	0.05	
PP-HF	0.0057	0.0011	5.0461	0.0000	
PP-MAPP	0.1660	0.0777	2.1369	0.0380	
HF-MAPP	0.0000	0.0000	65,535	0.5	
S = 4.53					
R-sq = 99.52%					
R-sq (adj) = 92.96%					

**Table 9:** ANOVA of factorial design for the Izod impact strength (I).

Source of Variation	Degree of Freedom (DF)	Sum of Squares (SQ)	Mean Squares (MQ)	F	Significance of F
Regression	5	3957.975	791.595	21.9522	4.4 $\times \;10^{-7}$
Residue	20	901.5002	45.075		
Total	25	4859.475			
**Term**	**Coeficients**	**Standard Error**	**Stat t**	**Value-** ***p***	
PP	0.1155	0.0300	3.8475	0.0010	
HF	0.0000	0.0000	65535	0.5	
MAPP	20.8182	11.9918	1.7360	0.05	
PP-HF	0.0035	0.0024	1.4600	0.1598	
PP-MAPP	−0.2836	0.1662	−1.7061	0.1035	
S = 6.71					
R-sq = 81.45%					
R-sq (adj) = 68.66%					

## Data Availability

The data presented in this study are available on request from the corresponding author.
